# Self-Education

**Published:** 1845-03

**Authors:** 


*Self-Education,*—Much less of success in life is, in reality, dependent upon accident, or what is called luck, than is commonly supposed. Far more depends upon the objects which a man proposes to himself; what attainments he aspires to; what is the circle which bounds his vision and thoughts; what he chooses, not to be *educated* for, but to *educate himself* for; whether he looks to the end and aim of the whole of life, or only to the present day or hour; whether he listens to the voice of indolence or vulgar pleasure, or to the stirring voice in his own soul, urging his ambition on to the laudable object.

